# Association of Peak Circulatory Power with Invasive Hemodynamic Severity in Pulmonary Hypertension

**DOI:** 10.3390/jcm15218309

**Published:** 2026-10-25

**Authors:** Ya Song, Chao Shi, Yan-Li Gu, Meng Li, Mig-Jing Zhao, Ting-Ting Dou, Liu Du, Ruo-Xi Li, Xue Feng, Xin-Xin Yan

**Affiliations:** State Key Laboratory of Cardiovascular Disease, National Center for Cardiovascular Diseases, Fuwai Hospital, Chinese Academy of Medical Sciences and Peking Union Medical College, 167 Beilishi Road, Xicheng District, Beijing 100037, China; songya@fuwai.com (Y.S.); 17612771720@163.com (C.S.);

**Keywords:** peak circulatory power, pulmonary hypertension, mixed venous oxygen saturation, hemodynamic severity, cardiopulmonary exercise testing

## Abstract

**Background:** Peak circulatory power (CP), derived from peak oxygen uptake per kilogram (peak VO_2_/kg) and peak systolic blood pressure (SBP), has prognostic value in cardiovascular disease, but its association with invasive hemodynamic severity in pulmonary hypertension (PH) remains unclear. We examined the relationship between peak CP and mixed venous oxygen saturation (SvO_2_) and other hemodynamic markers in patients with PH. **Methods:** This retrospective cross-sectional study included 133 adults with confirmed PH who underwent cardiopulmonary exercise testing and right heart catheterization before targeted therapy. Linear regression adjusted for age and sex evaluated associations between peak CP and SvO_2_, pulmonary vascular resistance (PVR), mean pulmonary arterial pressure (mPAP), N-terminal pro-brain natriuretic peptide (NT-proBNP), and six-minute walk distance (6MWD). Exploratory receiver operating characteristic (ROC) analysis assessed the ability of peak CP to identify SvO_2_ ≤ 65%. **Results:** Median age was 33 years, and 62.4% were female. After adjustment for age and sex, lower peak CP was associated with lower SvO_2_ (B per 100-unit increase = 0.30, 95% CI: 0.10–0.50, *p* = 0.011), higher PVR (B = −0.20, 95% CI: −0.40 to −0.10, *p* = 0.001), higher mPAP (B = −0.70, 95% CI: −1.10 to −0.20, *p* = 0.003), higher NT-proBNP (B = −19.0, 95% CI: −30.9 to −7.0, *p* = 0.002), and shorter 6MWD (B = 2.40, 95% CI: 0.50–4.30, *p* = 0.015). Peak CP identified SvO_2_ ≤ 65% (15/133, 11.3%) with an AUC of 0.781 (95% CI: 0.674–0.888), similar to peak VO_2_/kg (AUC 0.783). **Conclusions:** In patients with PH not receiving targeted therapy, lower peak CP is age- and sex-adjusted associated with lower SvO_2_, higher PVR, higher mPAP, higher NT-proBNP, and shorter 6MWD, suggesting that peak CP reflects exercise cardiovascular reserve and hemodynamic severity. Thresholds and discriminatory estimates require prospective validation.

## 1. Introduction

Pulmonary hypertension (PH) is a progressive pathophysiological disorder characterized by elevated pulmonary arterial pressure and increased pulmonary vascular resistance, ultimately leading to right heart failure and impaired exercise capacity [1]. Cardiopulmonary exercise testing (CPET) provides an integrated assessment of the cardiovascular, respiratory, and metabolic responses to exercise and has an established role in the evaluation of patients with PH [2].

Among CPET-derived variables, peak circulatory power (CP), defined as the product of peak oxygen uptake per kilogram (peak VO_2_/kg) and peak systolic blood pressure (SBP), has been proposed as a noninvasive surrogate of cardiac power during exercise [3]. Peak CP has been shown to carry prognostic value in patients with chronic heart failure [3,4], congenital heart disease [5], and coronary artery disease [6]. In patients with idiopathic pulmonary arterial hypertension (IPAH), Tang et al. reported that peak CP was a stronger predictor of cardiac events than peak VO_2_ or the ventilatory equivalent for carbon dioxide slope (VE/VCO_2_ slope) [7]. However, the relationship between peak CP and invasively measured hemodynamic markers of PH severity has not been fully characterized.

Mixed venous oxygen saturation (SvO_2_) is a key invasive hemodynamic variable that reflects the balance between systemic oxygen delivery and oxygen consumption. In PH, elevated pulmonary vascular resistance limits cardiac output augmentation during exercise, which reduces oxygen delivery and lowers SvO_2_ [8]. SvO_2_ is associated with disease severity and survival in pulmonary arterial hypertension (PAH) [9,10], although its incremental prognostic value beyond noninvasive risk stratification may vary across risk subgroups [11,12]. Because SvO_2_ requires invasive measurement, a noninvasive CPET-derived variable that is associated with SvO_2_ would be clinically relevant.

The present study therefore aimed to examine whether peak CP is associated with invasive hemodynamic severity in patients with confirmed PH, with a specific focus on SvO_2_. We also evaluated the associations between peak CP and other hemodynamic and functional markers, including pulmonary vascular resistance (PVR), mean pulmonary arterial pressure (mPAP), N-terminal pro-brain natriuretic peptide (NT-proBNP), and six-minute walk distance (6MWD). In addition, we explored the ability of peak CP to identify patients with SvO_2_ ≤ 65% in an exploratory analysis. We hypothesized that lower peak CP would be associated with more severe hemodynamic impairment in patients with PH.

## 2. Methods

### 2.1. Study Design and Population

This study adhered to the tenets of the Declaration of Helsinki and was approved by the Institutional Review Board and Ethics Committee of Fuwai Hospital, Beijing, China. Written informed consent was waived due to retrospective analysis of anonymized clinical data.

**Figure 1:**
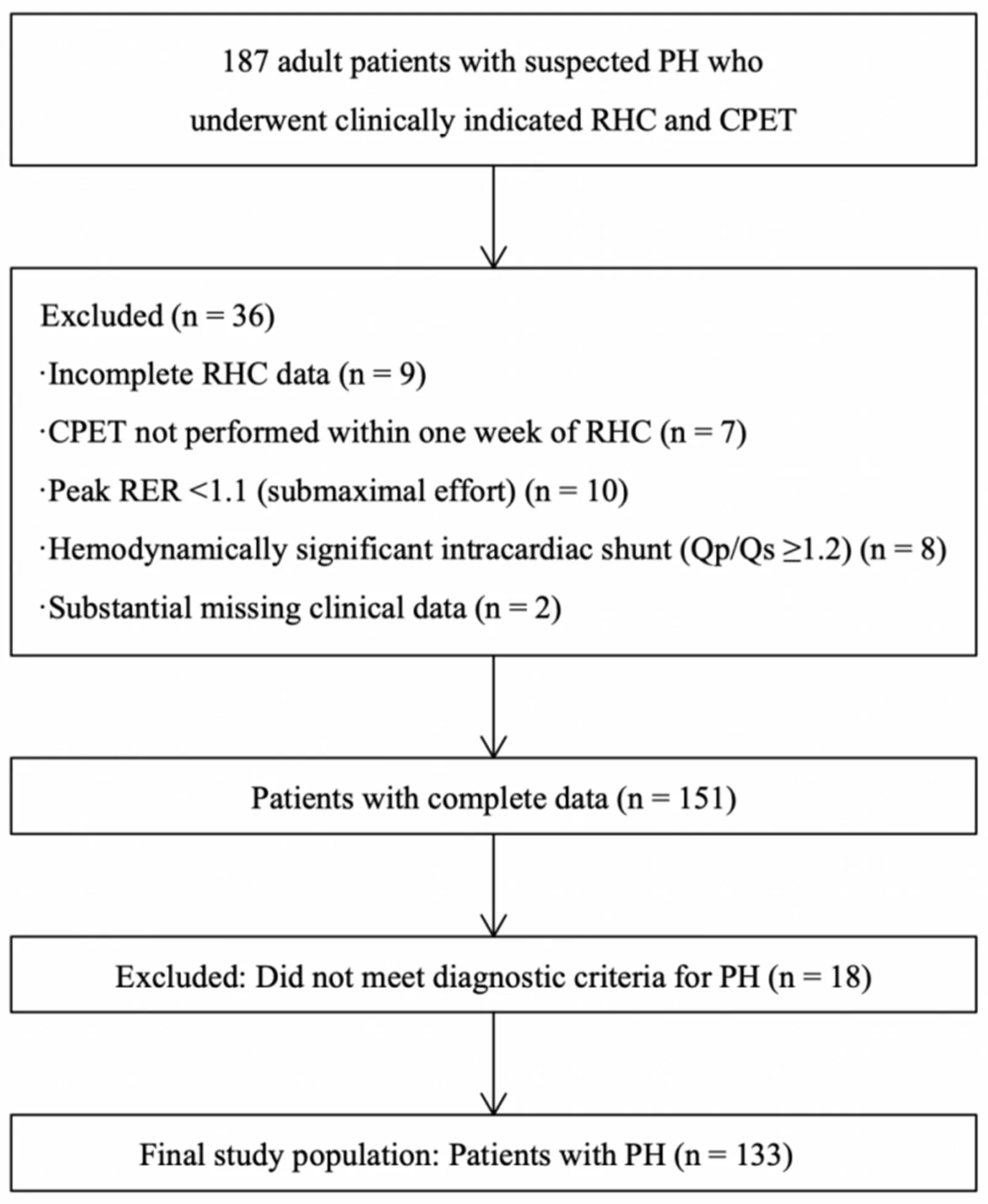
Study flow diagram. Of 187 adult patients with suspected pulmonary hypertension who underwent clinically indicated right heart catheterization (RHC) and cardiopulmonary exercise testing (CPET), 36 were excluded for the following reasons: incomplete RHC data (n = 9), CPET not performed within one week of RHC (n = 7), submaximal exercise effort defined as peak respiratory exchange ratio (RER) <1.1 (n = 10), hemodynamically significant intracardiac shunt defined as pulmonary-to-systemic flow ratio (Qp/Qs) ≥1.2 (n = 8), and substantial missing clinical data (n = 2). Among the remaining 151 patients, 133 were diagnosed with pulmonary hypertension and constituted the final study population.

This was a retrospective cross-sectional study. We screened 187 consecutive adult patients (≥18 years old) with suspected pulmonary hypertension who underwent clinically indicated right heart catheterization (RHC) and cardiopulmonary exercise testing (CPET) between March 2022 and May 2023. Patients were excluded for the following reasons: incomplete RHC data (*n* = 9), CPET not performed within one week of RHC (*n* = 7), submaximal exercise effort defined as peak respiratory exchange ratio (RER) < 1.1 (*n* = 10), hemodynamically significant intracardiac shunt defined as a pulmonary-to-systemic flow ratio (Qp/Qs) ≥ 1.2 on oximetry run (*n* = 8), and substantial missing clinical data (*n* = 2). Among the remaining 151 patients, 18 did not meet the diagnostic criteria for PH and were excluded from the present analysis. The final study population therefore consisted of 133 patients with confirmed pulmonary hypertension. The screening process is summarized in Figure 1.

### 2.2. Hemodynamic Definitions and PH Classification

Pulmonary hypertension was defined according to the 2022 European Society of Cardiology (ESC)/European Respiratory Society (ERS) guidelines as a resting mean pulmonary arterial pressure (mPAP) > 20 mm Hg measured during right heart catheterization. Pre-capillary PH was defined as mPAP > 20 mm Hg, pulmonary artery wedge pressure (PAWP) ≤ 15 mm Hg, and pulmonary vascular resistance (PVR) > 2 Wood units. Isolated post-capillary PH was defined as mPAP > 20 mm Hg, PAWP > 15 mm Hg, and PVR ≤ 2 Wood units, and combined post- and pre-capillary PH was defined as mPAP > 20 mm Hg, PAWP > 15 mm Hg, and PVR > 2 Wood units. Patients were classified into clinical groups according to the same guidelines: Group 1 (pulmonary arterial hypertension), Group 2 (PH associated with left heart disease), Group 3 (PH associated with lung diseases and/or hypoxia), Group 4 (chronic thromboembolic pulmonary hypertension and other pulmonary artery obstructions), and Group 5 (PH with unclear and/or multifactorial mechanisms) [1]. No patient in the present cohort was classified as Group 5.

### 2.3. Data Collection

Data were extracted from the electronic medical record system and included age, sex, height, weight, World Health Organization functional class (WHO-FC), N-terminal pro-brain natriuretic peptide (NT-proBNP), six-minute walk distance (6MWD), left ventricular ejection fraction (LVEF), comorbidities, clinical classification of PH, key parameters of CPET and RHC hemodynamics, and targeted therapy drugs documented at discharge. Patients with WHO-FC IV were not included because they were unable to achieve a peak RER ≥ 1.1, which was required for inclusion. No patient was receiving PAH-targeted therapy at the time of the index RHC and CPET; in the present cohort, PAH-targeted medications were initiated after the diagnostic evaluation and were recorded from discharge summaries.

### 2.4. Cardiopulmonary Exercise Testing

A standardized symptom-limited ramp protocol was performed using a bicycle ergometer (Ergoline, Bitz, Germany). The CPET gas analysis system (MasterScreen CPET; Jaeger Corp., Hoechberg, Germany) used a breath-by-breath system according to the American Thoracic Society/American College of Chest Physicians statement on CPET. Before each test, the equipment was calibrated according to the manufacturer’s specifications using reference and calibration gases. Standard 12-lead electrocardiograms (ECGs) and pulse oximetry were continuously monitored. Arterial blood pressure was measured every 2 min with an automatic cuff. Patients began with 3 min of rest and 3 min of unloaded cycling, followed by a progressively increasing workload of 10–25 watts/min in a ramp pattern to maximum tolerance and 6 min of recovery. Patients were asked to maintain a cadence of 55–65 revolutions per minute (rpm). The ramp protocol was individually selected so that subjects would reach their maximum tolerated workload within 8 to 12 min of exercise. Each participant breathed through a mask connected to a calibrated volume sensor, which measured oxygen uptake (VO_2_), carbon dioxide production (VCO_2_), and minute ventilation (VE). Patients were allowed to stop at any time if they experienced fatigue, dyspnea, chest tightness, or other forms of discomfort. Peak VO_2_ was determined as the average oxygen uptake over the last 30 s of exercise. The ventilatory equivalent for carbon dioxide slope (VE/VCO_2_ slope) was calculated by linear regression below the ventilatory compensation point. Peak circulatory power (CP) was calculated as the product of peak oxygen uptake per kilogram (peak VO_2_/kg) and peak systolic blood pressure (SBP). All included patients achieved a peak respiratory exchange ratio (RER) ≥ 1.1, indicating maximal effort.

### 2.5. Right Heart Catheterization

All patients underwent RHC in the supine position after an overnight fast. Hemodynamic measurements included right atrial pressure (RAP), mPAP, PAWP, PVR, cardiac output (CO), cardiac index (CI), and mixed venous oxygen saturation (SvO_2_). Pressures were recorded at end-expiration. In patients without intracardiac shunting, CO was measured by both thermodilution (average of three measurements) and the Fick method; the Fick-derived value was used when the difference between the two methods exceeded 10%. In patients with congenital heart disease or any suspected residual shunt, cardiac output was determined exclusively by the Fick method. SvO_2_ was measured from blood samples obtained in the pulmonary artery. Because patients with hemodynamically significant intracardiac shunting (Qp/Qs ≥ 1.2 on oximetry run) were excluded, pulmonary arterial blood was considered representative of true mixed venous blood for the SvO_2_ analyses.

### 2.6. Statistical Analysis

Continuous variables were expressed as mean ± standard deviation (SD) or median (25–75th percentile) depending on distribution. Normality was assessed using the Shapiro–Wilk test. Categorical variables are presented as counts and percentages.

Univariable associations between peak CP and markers of PH severity, including WHO functional class as an ordinal variable, were assessed using Spearman rank correlation.

The primary outcome was SvO_2_, and its association with peak CP was evaluated using univariable and multivariable linear regression. Secondary outcomes included PVR, mPAP, NT-proBNP, and 6MWD. All secondary analyses were exploratory, and no adjustment for multiplicity was performed.

Model assumptions were assessed using residual and Q-Q plots. For skewed outcomes (NT-proBNP and PVR), log-transformed analyses were additionally performed. (Supplementary Figure S1) Multivariable models were prespecified and adjusted only for age and sex to avoid overfitting. Regression coefficients were expressed per 100-unit increase in peak CP for clinical interpretability. No stepwise variable selection was performed. Multicollinearity was assessed using variance inflation factors; all values were <1.1, indicating no relevant collinearity.

Receiver operating characteristic (ROC) analysis was used to evaluate the ability of peak CP, peak VO_2_/kg, VE/VCO_2_ slope, and peak SBP to identify patients with reduced SvO_2_ (defined as SvO_2_ ≤ 65%, *n* = 15). We reported areas under the curve (AUC) with 95% confidence intervals (CIs) but did not derive optimal cut-off values because these analyses were hypothesis-generating only.

All statistical tests were two-sided, and *p* < 0.05 was considered statistically significant. Data management and analyses were performed using SPSS version 26.0 (IBM, Armonk, NY, USA).

## 3. Results

### 3.1. General Information

A total of 133 patients with confirmed PH were included in the final analysis (Figure 1). Baseline characteristics are summarized in Table 1. The median age was 33 years (IQR 27–42.5), and 83 patients (62.4%) were female. The majority of patients had Group 1 PAH (*n* = 112, 84.2%), most commonly associated with congenital heart disease (*n* = 86, 64.7%). Other Group 1 subtypes included idiopathic PAH (*n* = 22, 16.5%), PAH associated with connective tissue disease (*n* = 1, 0.8%), portopulmonary hypertension (*n* = 2, 1.5%), and heritable PAH (*n* = 1, 0.8%). Group 2 and Group 3 PH each accounted for 3 patients (2.3%), and Group 4 PH was present in 15 patients (11.3%), including 13 patients (9.8%) with chronic thromboembolic pulmonary hypertension (CTEPH) and 2 patients (1.5%) with other pulmonary artery obstructions. No patient was in WHO-FC IV, as all included patients were required to achieve a peak RER ≥ 1.1 during maximal CPET. Importantly, no patient was receiving PAH-targeted therapy at the time of RHC and CPET; targeted medications were initiated only after the diagnostic evaluation and were recorded at hospital discharge.

### 3.2. Correlations Between Peak CP and Markers of PH Severity

**Figure 2:**
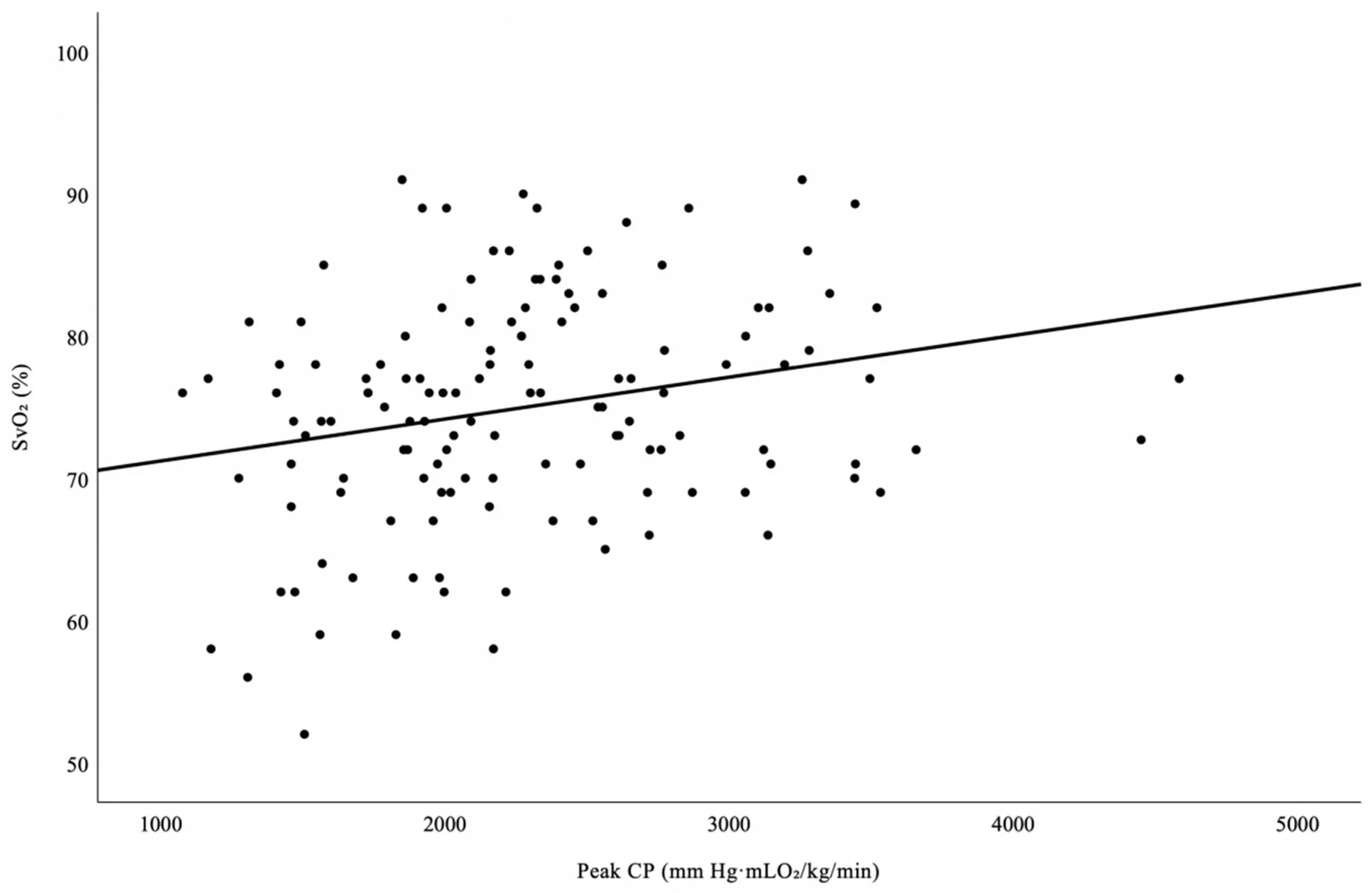
Scatter plot of peak CP versus SvO_2_. Scatter plot showing the relationship between peak circulatory power (CP) and mixed venous oxygen saturation (SvO_2_) in 133 patients with pulmonary hypertension. The solid line represents the linear regression fit. Peak CP correlated positively with SvO_2_ (r = 0.244, *p* = 0.005).

Peak CP correlated positively with SvO_2_ (rho = 0.244, *p* = 0.005), 6MWD (rho = 0.278, *p* = 0.001), and CO (rho = 0.206, *p* = 0.018), and negatively with PVR (rho = −0.326, *p* < 0.001), mPAP (rho = −0.235, *p* = 0.007), NT-proBNP (rho = −0.376, *p* < 0.001), WHO-FC (rho = −0.222, *p* = 0.010), and VE/VCO_2_ slope (rho = −0.175, *p* = 0.044) (Supplementary Table S1). No significant correlation was observed between peak CP and PETCO_2_ (rho = 0.117, *p* = 0.180). The relationship between peak CP and SvO_2_ is shown in Figure 2.

### 3.3. Age- and Sex-Adjusted Associations of Peak CP with Hemodynamic and Functional Markers

In multivariable linear regression adjusted for age and sex, with B coefficients expressed per 100-unit increase in peak CP, peak CP was associated with SvO_2_ (B = 0.30, 95% CI: 0.10 to 0.50, *p* = 0.011), PVR (B = −0.20, 95% CI: −0.40 to −0.10, *p* = 0.001), mPAP (B = −0.70, 95% CI: −1.10 to −0.20, *p* = 0.003), NT-proBNP (B = −19.0, 95% CI: −30.9 to −7.0, *p* = 0.002), and 6MWD (B = 2.40, 95% CI: 0.50 to 4.30, *p* = 0.015) (Table 2). For the skewed outcomes NT-proBNP and PVR, we also performed log-transformed analyses, and the associations remained significant and directionally consistent (Supplementary Table S2 and Figure S1). No relevant multicollinearity was observed (all VIF < 1.1).

### 3.4. Exploratory ROC Analysis

**Figure 3:**
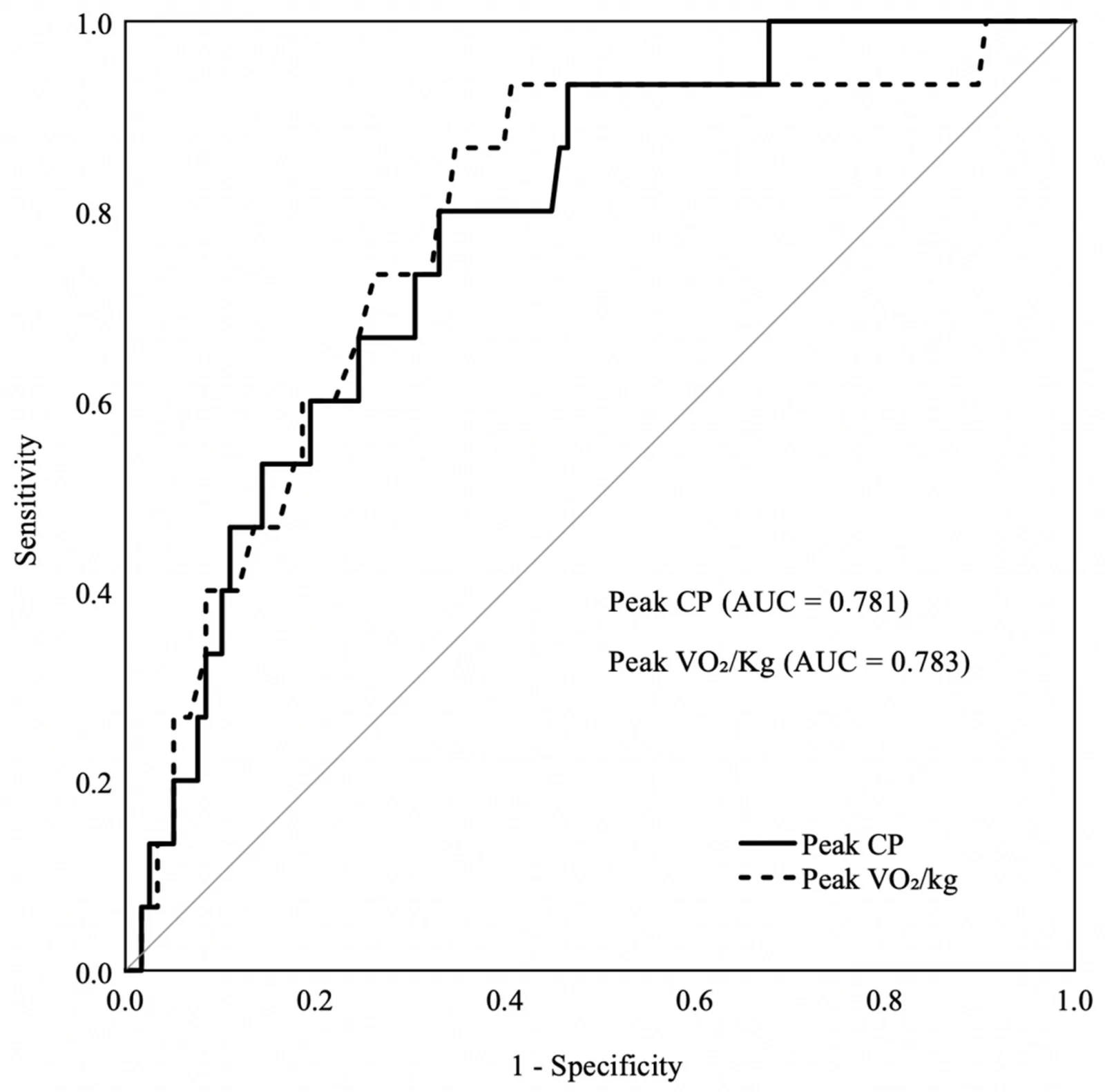
Receiver operating characteristic curves for identification of SvO_2_ ≤65%. Receiver operating characteristic (ROC) curves for peak circulatory power (CP) and peak oxygen uptake per kilogram (peak VO_2_/kg) in identifying patients with SvO_2_ ≤ 65%. The area under the curve (AUC) was 0.781 (95% CI: 0.674–0.888) for peak CP and 0.783 (95% CI: 0.663–0.903) for peak VO_2_/kg. The diagonal line represents chance discrimination (AUC = 0.5).

Fifteen of 133 patients (11.3%) had SvO_2_ ≤ 65%. In exploratory ROC analysis, peak CP identified patients with SvO_2_ ≤ 65% with an AUC of 0.781 (95% CI: 0.674–0.888). This was similar to peak VO_2_/kg (AUC 0.783, 95% CI: 0.663–0.903). The VE/VCO_2_ slope showed an AUC of 0.711 (95% CI: 0.571–0.851), and peak SBP showed an AUC of 0.646 (95% CI: 0.510–0.782) (Table 3; Figure 3; Supplementary Figure S2). No formal statistical comparison of AUCs was performed.

### 3.5. Sensitivity Analysis

In a sensitivity analysis restricted to patients with Group 1 PAH (*n* = 112), the association between peak CP and SvO_2_ remained directionally consistent. In univariable linear regression, peak CP was significantly associated with SvO_2_ (B = 0.003, 95% CI: 0.000–0.005, *p* = 0.020). After adjustment for age and sex, the association was borderline significant (B = 0.002, 95% CI: 0.000–0.004, *p* = 0.057). Spearman correlation showed a weak but significant positive correlation (rho = 0.199, *p* = 0.032). In exploratory ROC analysis, peak CP identified SvO_2_ ≤ 65% with an AUC of 0.745 (95% CI: 0.599–0.890, *p* = 0.015). These findings are summarized in Supplementary Table S3.

## 4. Discussion

After adjustment for age and sex, lower peak CP was associated with higher PVR, higher mPAP, higher NT-proBNP, lower SvO_2_, and shorter 6MWD in this cohort of patients with confirmed PH who were not receiving PAH-targeted therapy at the time of evaluation. No significant association was found between peak CP and cardiac output. These findings suggest that peak CP may reflect exercise cardiovascular reserve and hemodynamic severity in PH rather than resting right heart pressure or flow alone. In a sensitivity analysis restricted to Group 1 PAH, the association between peak CP and SvO_2_ remained directionally consistent, although it was borderline significant after adjustment for age and sex. Given the predominance of PAH associated with congenital heart disease, these findings should be extrapolated to other PH subtypes with caution.

The choice of SvO_2_ as the primary outcome was based on its physiological role as a direct marker of the balance between systemic oxygen delivery and oxygen consumption. SvO_2_ has been associated with disease severity and survival in PAH [9,10]. Khirfan et al. reported that SvO_2_ was a better prognosticator of transplant-free survival than CI in patients with PAH [9]. Current risk stratification models in PAH incorporate hemodynamic variables such as SvO_2_, which have been shown to improve prognostic assessment in both registry-based and clinical trial cohorts [13,14]. In the COMPERA 2.0 risk assessment model, SvO_2_ remains an important hemodynamic variable for stratifying patients into risk categories, with values > 65% generally considered favorable [15]. More recently, evidence suggests that the incremental prognostic value of SvO_2_ may be most evident in patients at intermediate risk according to noninvasive stratification, whereas its additional value in low- or high-risk subgroups is less clear [11,12]. In the present study, the association between peak CP and SvO_2_ therefore supports the hypothesis that CP carries information about oxygen delivery and circulatory reserve, although it does not establish interchangeability with invasive SvO_2_ measurement.

The associations between peak CP and invasive hemodynamic markers are biologically plausible. PVR is the main determinant of right ventricular afterload in PH [8]. In pulmonary hypertension, reduced SvO_2_ primarily reflects right ventricular–pulmonary arterial uncoupling, which leads to reduced stroke volume and low cardiac output, causing peripheral vasoconstriction and reduced tissue perfusion. When PVR is high, the right ventricle cannot adequately increase pulmonary blood flow during exercise, which limits peak VO_2_ and systemic pressure response, both of which are components of CP. This is consistent with the concept that right ventricular–pulmonary arterial coupling is a key determinant of exercise capacity in PH [16,17]. Vanderpool et al. showed that right ventricular–pulmonary arterial coupling predicts outcome in patients referred for PH [16]. Similarly, the inverse associations of CP with mPAP and NT-proBNP are consistent with the concept that CP may reflect increased pulmonary vascular load and neurohormonal activation [16,18,19], although these cross-sectional data support association rather than causal or incremental value, and further studies are needed to clarify the clinical implications. The positive association between CP and 6MWD further supports the relationship between CP and functional capacity. This is consistent with previous studies showing that CPET-derived exercise capacity correlates with 6MWD in PAH [20,21]. By contrast, the lack of association between peak CP and resting CO may reflect the fact that resting flows are influenced by compensatory mechanisms and do not fully capture the dynamic impairment that occurs during exercise. The abnormal exercise response in PH is characterized by reduced oxygen uptake, impaired ventilatory efficiency, and abnormal pulmonary hemodynamics [22]. In addition, peak CP was only weakly associated with the VE/VCO_2_ slope and was not significantly associated with end-tidal partial pressure of carbon dioxide (PETCO_2_). This pattern suggests that CP predominantly reflects circulatory rather than ventilatory impairment in PH.

Previous studies have shown that peak CP may have prognostic value in several cardiovascular populations. In chronic heart failure, Cohen-Solal et al. identified peak CP as a strong predictor of outcomes [3]. In adults with congenital heart disease, Giardini et al. reported that peak CP independently predicted prognosis [5]. Similarly, in children after the Fontan procedure, peak CP has been proposed as an indicator of clinical status [23]. In patients with coronary artery disease, peak CP has been characterized as a marker of functional significance [6]. In pulmonary arterial hypertension, Hirashiki et al. showed that peak CP may provide insights into the response to goal-oriented therapy [24], and Tang et al. reported that peak CP was a stronger predictor of cardiac events than peak VO_2_ or VE/VCO_2_ slope in patients with IPAH [7]. However, most prior studies have not directly examined the relationship between peak CP and invasively measured SvO_2_ in patients with established PH. The present study extends the literature by demonstrating that peak CP is associated with resting SvO_2_ and with multiple invasive hemodynamic markers, including PVR and mPAP, in a cohort in which CPET and right heart catheterization (RHC) were performed before initiation of targeted therapy. Furthermore, the finding that CP is not significantly related to PETCO_2_ and is only weakly related to the VE/VCO_2_ slope helps to position CP as a predominantly circulatory rather than ventilatory marker in this population [2].

As expected, peak CP was strongly correlated with peak oxygen uptake per kilogram (peak VO_2_/kg). In exploratory ROC analysis, peak CP did not demonstrate incremental discrimination over peak VO_2_/kg for identifying patients with SvO_2_ ≤ 65%; however, no formal statistical comparison of AUCs was performed, and this finding should be considered descriptive only. Then, this finding should be interpreted as evidence that CP and peak VO_2_/kg provide similar discriminatory information in this dataset, rather than as a reason to dismiss CP. Peak VO_2_ is a well-established prognostic marker in PAH [25], and recent studies have confirmed its prognostic value in contemporary PAH cohorts [26,27]. Because CP is mathematically derived from peak VO_2_ and peak SBP, a high correlation between CP and peak VO_2_/kg is expected. Nevertheless, CP may still provide a composite view of exercise circulatory reserve that is not fully captured by peak VO_2_ alone, because it integrates the systemic pressure response during exercise. However, the lack of formal statistical comparison between areas under the curve (AUCs) limits definitive conclusions about incremental value, and this issue should be examined in larger studies.

This study has several important limitations. First, the study was retrospective, cross-sectional, and single-center, which limits causal inference and generalizability. Second, the cohort was heterogeneous and dominated by Group 1 PAH, particularly PAH associated with congenital heart disease (CHD). Detailed anatomical data on specific congenital defects and repair status were not uniformly available, and patients with hemodynamically significant residual shunts were excluded. This may limit the applicability of the findings to more complex CHD populations and to other PH subtypes. Third, patients with World Health Organization functional class IV were excluded because they could not achieve a peak RER ≥ 1.1, which was an inclusion criterion. Consequently, the findings mainly apply to relatively exercise-capable patients with PH and may not be generalizable to the full spectrum of disease severity. Fourth, only 15 of 133 patients (11.3%) had SvO_2_ ≤ 65%, limiting the stability of the ROC analysis; in addition, the sensitivity analysis was restricted to patients with Group 1 PAH (*n* = 112), and the smaller sample size means these results should be considered supportive rather than definitive. Fifth, peak CP is mathematically derived from peak VO_2_ and peak SBP, which limits its ability to provide incremental information beyond these components. Sixth, no prospective external validation was performed, and no longitudinal outcomes were analyzed, so the prognostic and treatment-guiding implications of these findings remain to be determined. Finally, unmeasured confounders such as hemoglobin concentration and arterial oxygen saturation may have influenced the observed associations.

The present findings support the concept that peak CP is a noninvasive marker that tracks hemodynamic severity in patients with PH, particularly through its association with SvO_2_ and PVR. However, the results do not support the use of peak CP as a replacement for invasive hemodynamic assessment or as a standalone indicator for initiating or modifying therapy. Future prospective studies should evaluate whether changes in peak CP over time parallel changes in invasive hemodynamics and whether CP provides incremental value over established CPET variables such as peak VO_2_ and VE/VCO_2_ slope.

## 5. Conclusions

In patients with PH not receiving PAH-targeted therapy, lower peak CP was associated with lower SvO_2_, higher PVR, higher mPAP, higher NT-proBNP, and shorter 6MWD after adjustment for age and sex. These associations suggest that peak CP may reflect exercise cardiovascular reserve and hemodynamic severity in PH. These findings primarily reflect a PAH population enriched for congenital heart disease and require prospective validation in broader PH populations before clinical application.

## Figures and Tables

**Table 1:** Baseline characteristics.

Characteristics	PH Patients (*n* = 133)
Age, years	33 (27, 42.5)
Female, %	83 (62.4)
Height, cm	164.3 (7.2)
Weight, kg	59 (50, 68)
WHO-FC, III/I-II	14/119
6MWD, m	483.4 (73.2)
NT-proBNP, pg/ml	128 (84, 271)
LVEF, %	65.7 (4.9)
Systemic hypertension, %	12 (9.0)
Hypercholesterolemia, %	20 (15)
Hyperuricemia, %	12 (9.0)
GROUP 1 PAH, %	112 (84.2)
PAH idiopathic, %	22 (16.5)
PAH due to congenital heart disease, %	86 (64.7)
PAH due to connective tissue disease, %	1 (0.8)
PAH due to portal hypertension, %	2 (1.5)
PAH due to heritability, %	1 (0.8)
GROUP 2 PH associated with left heart disease, %	3 (2.3)
GROUP 3 PH associated with lung diseases, %	3 (2.3)
GROUP 4 PH associated with pulmonary artery obstructions, %	15 (11.3)
CTEPH, %	13 (9.8)
Other pulmonary artery obstructions, %	2 (1.5)
MAP, mmHg	84 (75.5, 89)
RAP, mmHg	5 (4, 7)
mPAP, mmHg	47 (36.5, 64)
PAWP, mmHg	8 (6, 11)
PVR, WU	7 (4, 11)
SvO_2_, %	75 (8)
CO, L/min	5.7 (4.5, 6.8)
CI, L/min/m^2^	3.3 (1)
Peak CP, mm Hg·mLO_2_/kg/min	2170 (1851.5, 2651.6)
Peak VO_2_/kg, mL/min/kg	16.9 (3.5)
VE/VCO_2_ slope	38.7 (33.9, 44.6)
Peak SBP, mmHg	130 (117, 148.5)
Peak RER	1.1 (1.1, 1.2)
P_ET_CO_2_, mmHg	28.2 (5.2)

Values are expressed as *n* (%), ratio, mean (SD), or median (25th to 75th percentiles). WHO-FC, World Health Organization functional class; 6MWD, six-minute walking distance; NT-proBNP, N-terminal pro-brain natriuretic peptide; LVEF, left ventricular ejection fraction; PH, pulmonary hypertension; PAH, pulmonary arterial hypertension; CHD, congenital heart disease; CTEPH, chronic thromboembolic pulmonary hypertension; MAP, mean arterial pressure; RAP, right atrial pressure; mPAP, mean pulmonary arterial pressure; PAWP, pulmonary arterial wedge pressure; PVR, pulmonary vascular resistance; SvO_2_, mixed venous oxygen saturation; CO, cardiac output; CI, cardiac index; CP, circulatory power; VO_2_/kg, oxygen uptake per kilogram; VE/VCO_2_, ventilatory equivalent for carbon dioxide; SBP, systolic blood pressure; RER, respiratory exchange ratio; P_ET_CO_2_, end-tidal partial pressure of carbon dioxide. No patient was receiving PAH-targeted therapy at the time of CPET and RHC; targeted medications were initiated after the diagnostic evaluation. All patients had no hemodynamically significant intracardiac shunt (defined as Qp/Qs < 1.2 on oximetry run). No patient was in WHO functional class IV, as these patients were unable to complete maximal cardiopulmonary exercise testing. Detailed anatomical data were not uniformly available for all patients with CHD-associated PAH.

**Table 2:** Multivariable linear regression analyses for the association of peak CP with markers of PH severity.

Dependent Variable	Unadjusted B (95% CI) per 100 Units CP	*p*	Adjusted B (95% CI) per 100 Units CP *	*p*
SvO_2_	0.30 (0.10 to 0.50)	0.005	0.30 (0.10 to 0.50)	0.011
PVR	−0.20 (−0.40 to −0.10)	0.002	−0.20 (−0.40 to −0.10)	0.001
mPAP	−0.60 (−1.00 to −0.10)	0.020	−0.70 (−1.10 to −0.20)	0.003
NT-proBNP	−19.4 (−31.3 to −7.4)	0.002	−19.0 (−30.9 to −7.0)	0.002
6MWD	2.60 (0.70 to 4.40)	0.007	2.40 (0.50 to 4.30)	0.015

* Adjusted for age and sex. CP, peak circulatory power; PH, pulmonary hypertension; SvO_2_, mixed venous oxygen saturation; PVR, pulmonary vascular resistance; mPAP, mean pulmonary arterial pressure; NT-proBNP, N-terminal pro-brain natriuretic peptide; 6MWD, six-minute walking distance; B, unstandardized regression coefficient; CI, confidence interval; B values are expressed per 100-unit increase in peak circulatory power. Each row represents a separate linear regression model with the listed variable as the dependent variable, peak circulatory power as the exposure of interest, and adjustment for age and sex. The adjusted model included age and sex as covariates. We observed no multicollinearity (all VIF < 1.1).

**Table 3:** Receiver operating characteristic analysis for identification of SvO_2_ ≤ 65%.

Variable	AUC	95% CI	*p* Value
Peak CP	0.781	0.674–0.888	<0.001
Peak VO_2_/kg	0.783	0.663–0.903	<0.001
VE/VCO_2_ slope	0.711	0.571–0.851	0.003
Peak SBP	0.646	0.510–0.782	0.035

CP, circulatory power; VO_2_/kg, oxygen uptake per kilogram; VE/VCO_2_, ventilatory equivalent for carbon dioxide; SBP, systolic blood pressure; AUC, area under the receiver operating characteristic curve; CI, confidence interval. The outcome was defined as mixed venous oxygen saturation (SvO_2_) ≤ 65%. For peak CP, peak VO_2_/kg, and peak SBP, lower values indicated a higher likelihood of low SvO_2_; for the VE/VCO_2_ slope, higher values indicated a higher likelihood. Because of tied values in some variables, AUC estimates may be slightly biased; these analyses are exploratory and require external validation.

## Data Availability

The data presented in this study are available within the article. The raw datasets generated and/or analyzed during the current study are not publicly available due to ethical and privacy restrictions (the data contain information that could compromise the privacy of research participants) but are available from the corresponding author upon reasonable request.

## Supplementary Materials

The following supporting information can be downloaded at: https://www.mdpi.com/article/10.3390/jcm15218309/s1, Table S1: Univariate Spearman correlations between peak circulatory power and markers of pulmonary hypertension severity. Table S2: Log-transformed linear regression analyses for the association of peak circulatory power with NT-proBNP and PVR. Table S3: Sensitivity analysis restricted to patients with Group 1 PAH (n = 112). Figure S1: ROC curves for VE/VCO_2_ slope and peak SBP in identifying SvO_2_ ≤ 65%. Figure S2: Regression diagnostic plots for the linear regression models.

## Abbreviations

The following abbreviations are used in this manuscript:

PH	Pulmonary hypertension
CP	Peak circulatory power
SvO_2_	Mixed venous oxygen saturation
PVR	Pulmonary vascular resistance
mPAP	Mean pulmonary arterial pressure
NT-proBNP	N-terminal pro-brain natriuretic peptide
6MWD	Six-minute walking distance
SBP	Systolic blood pressure
CPET	Cardiopulmonary exercise testing
IPAH	Idiopathic pulmonary arterial hypertension
VE/VCO_2_ slope	Ventilatory equivalent for carbon dioxide slope
PAH	Pulmonary arterial hypertension
RHC	Right heart catheterization
RER	Respiratory exchange ratio
Qp/Qs	Pulmonary-to-systemic flow ratio
ESC	European Society of Cardiology
ERS	European Respiratory Society
PAWP	Pulmonary artery wedge pressure
WHO-FC	World Health Organization functional class
LVEF	Left ventricular ejection fraction
ECG	Electrocardiogram
VO_2_	Oxygen uptake
VCO_2_	Carbon dioxide production
VE	Minute ventilation
RAP	Right atrial pressure
CO	Cardiac output
CI	Cardiac index
SD	Standard deviation
ROC	Receiver operating characteristic
AUC	Area under the curve
CI	Confidence interval
VIF	Variance inflation factor
IQR	Interquartile range
CTEPH	Chronic thromboembolic pulmonary hypertension
CHD	Congenital heart disease
PETCO_2_	End-tidal partial pressure of carbon dioxide
