# Supplementary material for: Association of Peak Circulatory Power with Invasive Hemodynamic Severity in Pulmonary Hypertension
Source: J Clin Med. 2026 Oct 25;15(21):8309. doi: 10.3390/jcm15218309 (PMC13764259; doi:10.3390/jcm15218309)
Supplement: Supplementary file 1 [file jcm-15-08309-s001.zip › jcm-4488892-supplementary.pdf]

Supplementary Table S1. Univariate Spearman correlations between peak circulatory power and markers of pulmonary hypertension severity.

**Variable**	**Spearman rho**	**p value**
SvO_2	0.244	0.005
PVR	-0.326	<0.001
mPAP	-0.235	0.007
NT-proBNP	-0.376	<0.001
CO	0.206	0.018
WHO functional class	-0.222	0.010
6MWD	0.278	0.001
$P_{ET}CO_2$	0.117	0.180
VE/VCO_2 slope	-0.175	0.044

Values are Spearman rank correlation coefficients. N = 133. Abbreviations: SvO_2, mixed venous oxygen saturation; PVR, pulmonary vascular resistance; mPAP, mean pulmonary arterial pressure; NT-proBNP, N-terminal pro-brain natriuretic peptide; WHO-FC, World Health Organization functional class; 6MWD, six-minute walking distance; CO, cardiac output; $PETCO_2$, end-tidal partial pressure of carbon dioxide; VE/VCO_2, ventilatory equivalent for carbon dioxide.

Supplementary Table S2. Log-transformed linear regression analyses for the association of peak circulatory power with NT-proBNP and PVR.

Dependent variable (log-transformed)	Model	Standardized β	p value
log_NTproBNP	Unadjusted	-0.387	<0.001
log_NTproBNP	Adjusted for age and sex	-0.356	<0.001
log_PVR	Unadjusted	-0.321	<0.001
log_PVR	Adjusted for age and sex	-0.345	<0.001

N = 133. Log-transformed analyses were performed because NT-proBNP and PVR were skewed. After log-transformation, residuals were normally distributed (standardized residuals ranged from -2.46 to 2.54 for log_NTproBNP and -2.53 to 2.55 for log_PVR), with no influential outliers (all Cook's distances <0.1). NT-proBNP, N-terminal pro-brain natriuretic peptide; PVR, pulmonary vascular resistance.

Supplementary Table S3. Sensitivity analysis restricted to patients with Group 1 PAH (n = 112).

Analysis	Statistic	Value	95% CI	p value
Univariable linear regression (dependent: SvO_2)	B for peak CP	0.003	0.000–0.005	0.020
Multivariable linear regression (adjusted for age and sex)	B for peak CP	0.002	0.000–0.004	0.057
Spearman correlation	rho	0.199	—	0.032
ROC analysis (SvO_2 ≤65%)	AUC	0.745	0.599–0.890	0.015

N = 112. AUC, area under the curve; CI, confidence interval; CP, circulatory power; ROC, receiver operating characteristic; SvO_2, mixed venous oxygen saturation. The multivariable model was adjusted for age and sex.

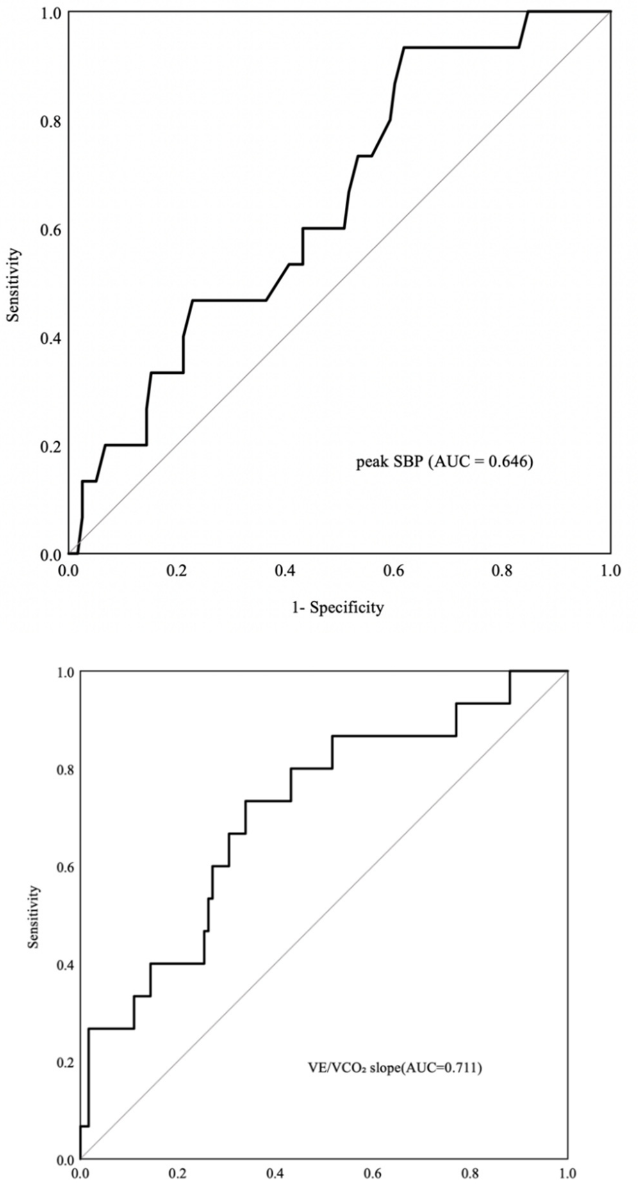

Supplementary Figure S1. ROC curves for VE/VCO_2 slope and peak SBP in identifying SvO_2 ≤65%.

ROC, receiver operating characteristic; AUC, area under the curve; VE/VCO_2, ventilatory equivalent for carbon dioxide; SBP, systolic blood pressure; SvO_2, mixed venous oxygen saturation. Note: For peak CP, peak VO_2/kg, and peak SBP, lower values indicate a higher likelihood of low SvO_2; for the VE/VCO_2 slope, higher values indicate a higher likelihood.

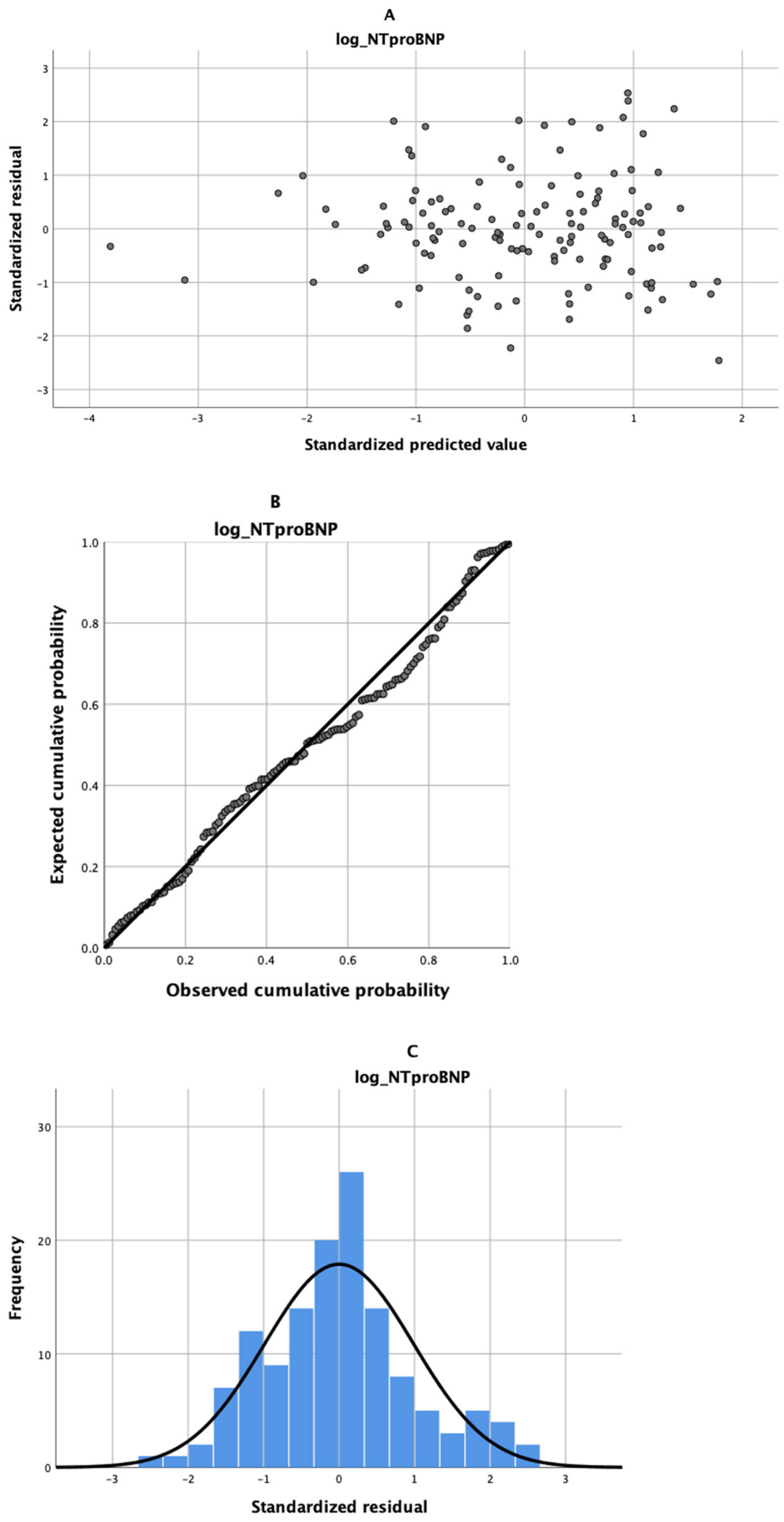
A
log_NTproBNP
Standardized residual
Standardized predicted value
B
log_NTproBNP
Expected cumulative probability
Observed cumulative probability
C
log_NTproBNP
Frequency
Standardized residual

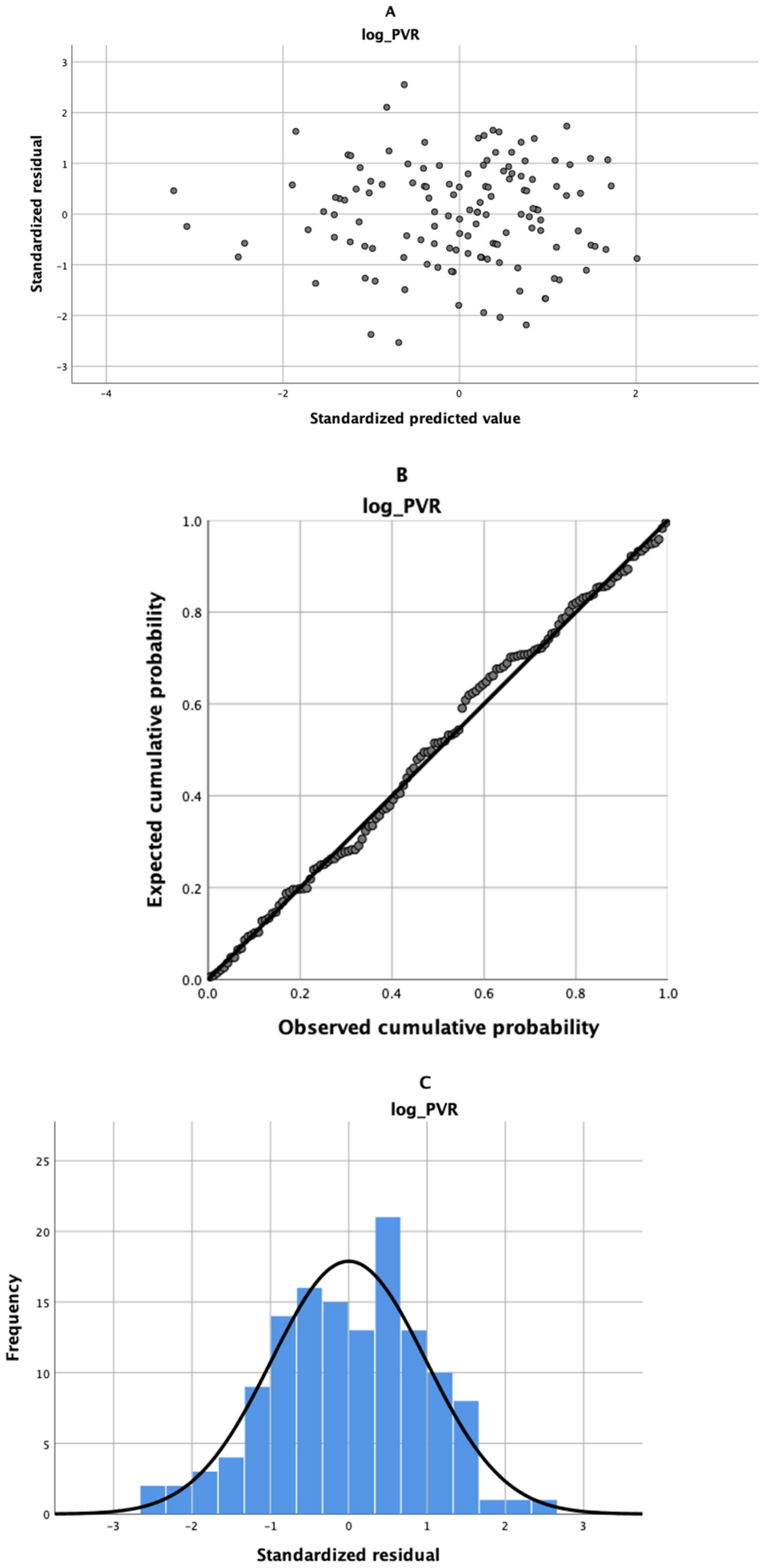

Supplementary Figure S2. Regression diagnostic plots for the linear regression models.

Regression diagnostic plots for the linear regression models. **(A)** Scatterplot of standardized residuals versus standardized predicted values. **(B)** Normal P-P plot of standardized residuals. **(C)** Histogram of standardized residuals. Panels show the models with SvO_2, log-transformed NT-proBNP, and log-transformed PVR as dependent variables, each adjusted for age and sex. No major violations of linearity, homoscedasticity, or normality were observed.
